# Psychopathic Traits and Their Relationship with the Cognitive Costs and Compulsive Nature of Lying in Offenders

**DOI:** 10.1371/journal.pone.0158595

**Published:** 2016-07-08

**Authors:** Bruno Verschuere, Willem in ´t Hout

**Affiliations:** 1 Department of Clinical Psychology, University of Amsterdam, Amsterdam, The Netherlands; 2 Forensic Psychiatric Center ´de Oostvaarderskliniek´, Almere, The Netherlands; Brain and Spine Institute (ICM), FRANCE

## Abstract

The cognitive view on deception holds that lying typically requires additional mental effort as compared to truth telling. Psychopathy, however, has been associated with swift and even compulsive lying, leading us to explore the ease and compulsive nature of lying in psychopathic offenders. We explored the costs of instructed lying versus truth telling through RTs and error rates in 52 violent male offenders, who were assessed with the Youth Psychopathic Traits Inventory (YPI). Our deception paradigm also included trials with the free choice to lie or tell the truth. By coupling monetary loss to slow and erroneous responding, we hypothesized that the frequency of lying despite likely negative consequences, would provide an index of compulsive lying. Offenders were slower and erred more often when lying than when telling the truth, and there was no robust association between psychopathy and the cognitive cost of lying. From an applied perspective, this suggests that psychopathy may not threaten the validity of computerized cognition-based lie detection. In the face of probable negative consequences, high grandiose-manipulative offenders chose to lie three times as often as low grandiose-manipulative offenders. Our new lying frequency index is a first attempt to create a much needed tool to empirically examine compulsive lying, and provides preliminary support for the compulsive nature of lying in grandiose-manipulative offenders. Alternative interpretation of the findings are discussed.

## Introduction

“No man has a good enough memory to be a successful liar”

― Abraham Lincoln

“If you tell the truth, you don't have to remember anything.”

― Mark Twain

The quotes from Mark Twain and Abraham Lincoln point to the idea that the truth comes naturally, and that lying requires mental effort. In the last two decades, partly related to rise of neuroimaging techniques, there has been a great increase in studies that empirically tested the idea that lying is cognitively more demanding than truth telling. The first fMRI study on deception [1] scanned 10 intelligent young males while asked to make a speeded decision on simple Yes/No questions regarding activities that they may have performed that day (e.g., Drunk coffee?). Lying was associated with greater activity in the bilateral ventrolateral prefrontal cortices, medial prefrontal and premotor cortices, and left inferior parietal and lateral premotor cortices than truth telling. It also took participants considerably longer—about 200ms—to lie than to tell the truth. Both the increased prefrontal activity (for a meta-analysis see [2]) and the delay in responding (for a review see [3]) have been replicated by several studies. People also subjectively experience lying to be more difficult than telling the truth ([4], [5]). Thus, there is considerable support for the notion that the truth *typically* comes naturally and that lying *typically* requires additional effort (for reviews see [6] and [7]). The next step in the theoretical development of the cognitive view on deception is to map its boundary conditions. The current study explored the cognitive costs and pathological nature of lying in psychopathic offenders. Is lying also mentally taxing for psychopathic individuals or may lying be actually their natural response?

The Psychopathy Checklist—Revised (PCL-R, [8]) defines psychopathy in terms of shallow affect, a deceitful, dominant, and narcissistic interpersonal style, an irregular, irresponsible and antisocial lifestyle. Psychopathic individuals are often portrayed as naturally born liars, with lying and manipulating seen as core features of psychopathy. Despite differences in the conceptualization of psychopathy, deceitfulness is adopted as a key feature by many clinicians and scholars. Robbins ([9], pp. 157), for instance, argued that childhood predictors of adult psychopathy involved those who ´lied gratuitously, and showed little guilt over their behavior´. Consequently, the PCL-R includes ´conning/manipulative´, and even ´pathological lying´ as defining features of psychopathy. Likewise, a well-validated self-report measure of psychopathy, the Youth Psychopathic Traits Inventory (YPI; [10]) has three scales that directly assess deceptive behavior: ´Dishonest Charm´, ´Lying´, and ´Manipulation´.

The association between psychopathy and deception has been investigated with a variety of paradigms including self-report questionnaires, validity tests, laboratory cheating paradigms, file reviews, and lie detection tests (e.g., polygraph tests). Surprisingly, an early review of the literature by Hare and colleagues [11] found that the data on the relation between psychopathy and deception was very mixed. The research literature since then has continued to provide inconsistent support for the idea that psychopathic individuals would be particularly apt at lying (e.g., [12–20]) and seems to support Hare and colleagues’ [11] conclusion of ´a marked discrepancy between these empirical findings and clinical/behavioral manifestations of deception by psychopaths´. While psychopathic individuals are often portrayed as naturally born liars, there is only weak and inconsistent empirical evidence that psychopathic individuals would lie frequently, easily, and compulsively.

Clinical descriptions, psychopathy theory, and assessment instruments consider lying, manipulation and deceit to be at the heart of psychopathy, but empirical data do not provide unequivocal support for that notion. There are only very few studies, however, that examined the ease of lying in forensic samples. Note that the very notion of non-criminal psychopathy is debated (for reviews see [21], [22]), and that the range of psychopathy levels in non-forensic samples may be restricted, limiting the chance to observe psychopathy-related characteristics. We therefore examined the relation between psychopathy and the ease of lying in an offender sample. Moreover, we adjusted the Sheffield Lie Test [1] to also assess compulsive lying, by (1) including trials on which they were given the free choice to lie or tell the truth, and (2) coupling monetary loss to slow and erroneous responding (which is more likely to occur for lying than for truth telling; [1]). We hypothesized that the frequency of lying despite likely negative consequences, would provide an index of compulsive lying. As such, we aimed to assess, in an offender sample, whether psychopathy is related to smooth and compulsive lying.

## Method

The study was approved by the local ethical committee of the Psychology Department of the University of Amsterdam (2013-CP-3043). All participants provided written informed consent using a consent form approved by the local ethical committee. The script of the experimental task as well as the data are publically available on https://osf.io/w746e/

### Participants

Fifty-two violent male offenders were recruited in three Dutch forensic institutions. Exclusion criteria were (1) schizophrenia, bipolar disorder or acute psychotic symptoms, (2) clinician estimated IQ < 80, (3) visual impairment, 4) use of antipsychotic medication, and 5) somatic impairments effecting muscle- or coordination functions (e.g., Klinefelter).

On instructed lie and truth trials, participants nearly always (98%, *SD* = 4%) correctly indicated whether they were about to lie or tell the truth. One participant indicated on 24% of the instructed lie and truth trials that he would behave different from what was instructed (e.g., reported he would lie when instructed to tell the truth). We excluded the data of this participant, leaving *n* = 51. We also excluded the data of one participant because of an excessive error percentage on the instructed lie and truth trials (i.e., 52% errors; deviating more than 2.5SDs sample *M*: 23%, *SD* = 10%), leaving *n* = 50.

The final sample consisted of 50 patients that were on average 42 years old (*SD* = 12, *range* 23–67 years). Most patients had the Dutch nationality (90%), and were unmarried (87.5%). None had higher education at the college or university level. YPI was available for all patients and showed substantial variation across the psychopathy spectrum (*M*_YPI Total score_ = 84.24; *SD* = 17.83; range: 59–129), and WAIS-IQ was available for 43 patients (*M*_WAIS Total IQ score_ = 95.60; *SD* = 14.24; range: 64–131).

### Procedure

After providing written informed consent, participants completed the YPI and the modified Sheffield lie test in a session of about 1.5 hours that also included other questionnaires (i.e., The Experiences in Close Relationships questionnaire; [23]) and two computerized attention tasks. Participants received fixed financial compensation for their participation plus an amount that varied between €0 and €5, depending on their performance in the modified Sheffield lie test.

### Measures

#### Youth Psychopathic Traits Inventory

The *Youth Psychopathic Traits Inventory* (YPI; [10] Dutch version, [24]) is a self-report psychopathy scale scored in a 4-point Likert-scale ranging from 1 (does not apply at all) to 4 (applies very well). The 50 items load on 10 scales with the *Dishonest Charm*, *Grandiosity*, *Manipulation*, and *Lying* scales tapping into the factor Grandiose-manipulative (20 items; e.g., “I have the ability to con people by using my charm and smile”), the *Remorseless*, *Unemotionality*, and *Callousness* scales tapping into the factor Callous-unemotional (15 items; e.g., “I think that crying is a sign of weakness, even if no one sees you”), and the *Thrill-Seeking*, *Impulsiveness*, and *Irresponsibility* tapping into the factor Impulsive-irresponsible traits (15 items; e.g., "I consider myself as a pretty impulsive person"). The YPI items are worded in a positive direction in order to eliminate the effect of social desirability. The YPI was initially developed for use in adolescents, but has no adolescent-specific items, and has been shown to have good psychometric properties in young adults (e.g., [23]; [13]) as well as in adulthood [25]. In the present sample, the internal consistency (Chronbach´s *α*) was good for YPI Grandiose-manipulative (*α* = .89), YPI Callous-unemotional (*α* = .78), YPI Impulsive-irresponsible (*α* = .84), and the YPI total score (*α* = .91).

#### Modified Sheffield lie test

The *modified Sheffield lie test* was programmed with Inquisit software [26], which also measured RTs with millisecond accuracy.

In the *modified Sheffield lie test* (original version [1]), questions were presented with either the instruction to lie, the instruction to tell the truth, or the choice to lie or tell the truth. A cue preceding the questions informed participants to tell the truth (the letter W for ´Waarheid´ [Truth]), lie (the letter L for ´Liegen´ [Lie]) or chose to lie or tell the truth (the? symbol). Using their left hand, participants first pressed the Truth (the a-key) or Lie (the s-key) button to indicate that they would tell the truth or lie to the upcoming question. This self-paced button press was needed to disentangle errors from lies on the choice trials, and allowed to check adherence to the instructions. The question appeared on average 2s (range 1.5s to 2.5s) after pressing the lie or truth button. Questions appeared in the middle of the screen with the YES and NO reminders labels appearing below left and below right, respectively. Participants were instructed to answer the questions as fast as possible with their right hand, using the YES (the k-key) and NO (the l-key) buttons.

There were 40 questions: 20 questions required a YES response when telling the truth (e.g., Is it 2014?), and 20 questions required a NO response when telling the truth (e.g., Is it 2004?). Questions were tailored to assure that they required a YES or NO response. For instance, as data collection started in 2013 and ended in 2015, the question ´Is it 2014?´ was adjusted to ´Is it 2015?´ for participants tested in 2015.

Participants started this task with €5, and were informed that they would lose €0.05 for behavioral errors (e.g., pressing NO to a true question when instructed to tell the truth), and €0.20 for slow responses (i.e., RTs above 2s; participants were not informed on the exact response deadline). RTs on the cued lie and cued truth trials served as an index of the cognitive costs of lying. Reasoning that lying would be cognitively more demanding than truth telling and that fast and slow responding resulted in financial loss, lying frequency on the choice trials was taken as an index of compulsive lying (lying despite negative consequences).

The task was practiced in two blocks of 12 trials, using another set of very simple true (e.g., Is fire hot?) and false (e.g., Is ice warm?) questions. The first practice block had a fixed order that built up in complexity. The second practice block had a random order. After the two practice blocks, there was one block with 120 test trials: 40 instructed truth trials, 40 instructed lie trials, and 40 choice trials, presented in random order. Across individuals, but not within each individual, each question was presented about equally often with each of the 3 instruction cues. In sum, the *modified Sheffield lie test* consisted of a total of 142 trials: 24 practice trials (not taken into the analyses) and 120 test trials (taken into the analyses).

## Results

### Analyses

Because taxometric analyses indicate that psychopathy is a dimensional construct (see e.g., [27]), we used correlational analyses. The primary analyses involve Pearson correlations between psychopathy (YPI; see Supporting Information) on the one hand, and the ease of lying (RT_LIE_ minus RT_TRUTH_; Error_LIE_ minus Error_TRUTH_) and compulsivity of lying (lying frequency on the choice trials) on the other hand. We did not control for age or IQ in these analyses, because the psychopathy total and factor scores were unrelated to both age and IQ; all *r´s* <.13, all *p´s* > .40.

#### Modified Sheffield lie test

Participants erred more often when lying (*M* = 32%; *SD* = 13%) than when telling the truth (*M* = 18%; *SD* = 10%) on the instructed lie and truth trials, *t*(49) = 7.90, *p* < .001. We calculated the effect size for within-subject contrast as Cohen´s *d*_*within*_ = *M(Error*_*LIE*_ − *Error*_*TRUTH*_ / √(*SD*_*LIE*_^*2*^ + SD_*TRUTH*_^2^ − 2*r*SD_LIE_*SD_*TRUTH*_), where *r* is the correlation between *Error*_*LIE*_ and *Error*_*TRUTH*_ (here: *r* = .42). The difference between lying error rate and truth error rate was large *d*_*within*_ = 1.11 (95% CI: .74–1.47). We used (*Error*_*LIE*_ minus *Error*_*TRUTH*_) as a first index of the cognitive cost of lying.

Behavioral errors were excluded for RT analyses. To reduce the impact of outliers, we censored the RTs following established procedures ([28]; [29]). Specifically, we converted outlying RTs (2.40% of the data) below 300ms to 300ms and RTs above 3000ms to 3000ms (note that excluding rather than censoring outliers did not change the results). Participants were slower when lying (*M* = 1525ms; *SD* = 284) than when telling the truth (*M* = 1422; *SD* = 250) on the instructed lie and truth trials, *t*(49) = 5.52, *p* < .001. Controlling for the correlation between RT_LIE_ and RT_TRUTH_, *r* = .89, the difference between RT_LIE_ and RT_TRUTH_ was moderate to large *d*_*within*_ = 0.79 (95% CI: .45–1.38). We used (*RT*_*LIE*_ minus *RT*_*TRUTH*_) as a second index of the cognitive cost of lying.

**Fig 1:**
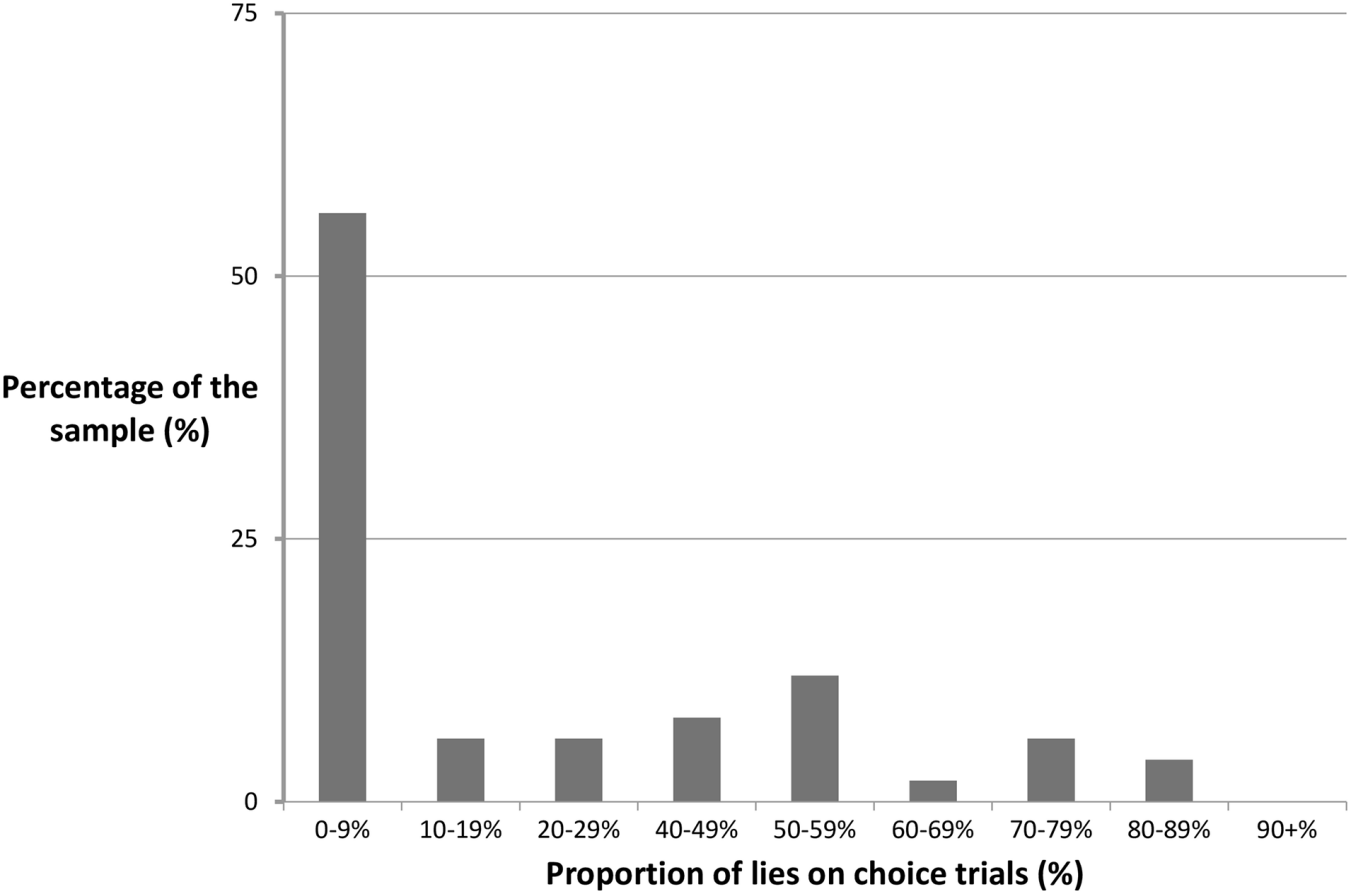
Frequency of lying (choice trials).

Participants mostly chose to tell the truth on the choice trials, as evidenced by the low average lying frequency (*M* = 22%, *SD* = 28%). Forty-two percent of the sample never lied on the choice trials. There was considerable variance, and Fig 1 illustrates that lying frequency ranged from 0% to 83%. Lying frequency on the choice trials was used as an index of compulsive lying.

#### Psychopathy and lying

Table 1 plots the association between psychopathy on the one hand, and the cognitive cost of lying (*Error*_*LIE*_ minus *Error*_*TRUTH*_; and *RT*_*LIE*_ minus *RT*_*TRUTH*_) and compulsive lying (lying frequency on the choice trials) on the other hand.

**Table 1:** Correlation matrix displaying the associations between psychopathy and the cognitive costs of lying (in RTs and errors) and lying frequency on the choice trials.

	Psychopathy				Deception Indices		
	YPIGM	YPICU	YPIII	YPITOT	RT_LIE_-RT_TRUTH_	Errors_LIE_-Errors_TRUTH_	Choice to lie
YPI-GM	-	.47**^a^	.69***	.91***	-.17	-.03	.35*
YPI-CU		-	.24	.66***	-.22	-.29*	.13
YPI-II			-	.83**	.10	.09	.17
YPI-TOT				-	-.11	-.07	.28
RT_LIE_-RT_TRUTH_					-	.31*	-.38**
Errors_LIE_-Errors_TRUTH_						-	-.31*

^a^ Significant effects with *p*< .05 are designated as (*), effects with *p* < .01 are designated as (**), and effects with *p* < .001 are designated as (***). No correction for multiple testing was applied, and effect sizes are more important than significance levels (*r*´s can be labelled as small, moderate, and large effects, from .1, .3, and .5 onwards, respectively)

First, there appeared an association between callous-unemotional traits (YPI-CU) and the cost of lying in error rates, *r* = -.29, *p* = .04 that indicated a reduce cost of lying in high callous-unemotional offenders. This association, however, was carried by two outliers with YPI-CU scores (i.e., 45) that deviated more than 3SDs from the sample mean (i.e., *M* = 25.92, *SD* = 6.09). After excluding the two outliers, the correlation was no longer significant, *r* = -.22, *p* = .13. We therefore think that the observed association was distorted by the two outliers, and that there is no real association between callous-unemotional traits (YPI-CU) and the cost of lying in error rates.

**Fig 2:**
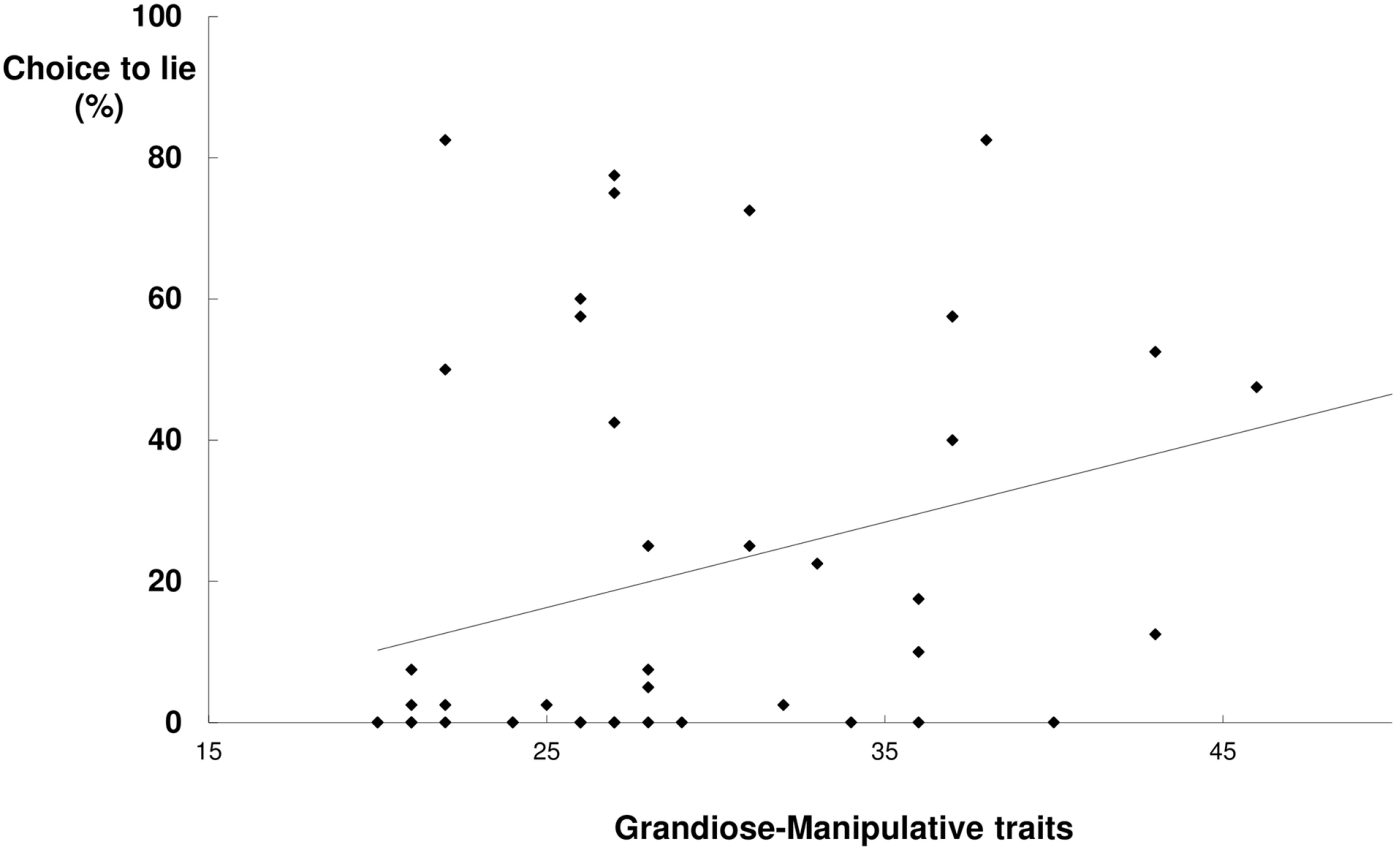
Grandiose-Manipulative traits and frequency of lying (choice trials).

Second, the more grandiose-manipulative (YPI-GM), the more frequent the choice to lie, *r* = .35, *p* = .01, see Fig 2. That correlation was not affected by the exclusion of possible outliers (i.e., YPI-GM scores that deviated more than 2.5SDs from the M). To illustrate the size of the difference, we compared lying frequency in the lowest thirtile (YPI-GM < = 25; *n* = 15) versus the highest thirtile (YPI-GM > = 32; *n* = 16), and found that high GM offenders (*M* = 31.09, *SD* = 26.16) chose to lie three times as often as low GM offenders (*M* = 9.83, *SD* = 23.80). This was also the case when comparing the lowest quartile (YPI-GM < = 22; *n* = 12) versus the highest quartile (YPI-GM > = 36; *n* = 13) with a lying frequency of *M* = 36.34 (*SD* = 25.89) versus *M* = 12.08 (*SD* = 26.32).

The deception indices appeared not completely independent from each other: The higher the cognitive cost of deception in error rates, the higher the cognitive cost of deception in RTs, *r* = .31, *p* = .03. Also, the more frequent the choice to lie, the lower the cognitive cost of deception (Errors: RTs: *r* = -.33, *p* = .02; RTs: *r* = -.38, *p* = .01).

## Discussion

Laboratory evidence in undergraduates has fuelled the idea that the truth typically comes naturally, and that lying requires additional mental effort. This cognitive view on deception has gained wide attention and led to the development of cognition-based lie detection techniques [7]. An important next step is to map the boundary conditions of the cognitive theory on deception. In the present study, we investigated the relationship between psychopathy and the cognitive costs and the compulsive nature of lying.

### Choosing to lie

When provided with the choice to lie versus tell the truth, we found that offenders preferred to tell the truth, and chose to lie on only 22% of the trials. An important restriction to this finding is that a penalty was coupled to slow and erroneous responding, indirectly deterring from chosing to lie. This payment system was set-up to create an index of pathological lying (i.e., lying despite negative consequences). Taking this important restriction in mind, the fact that offenders preferred truth telling above lying, supports the cognitive theory of lying. Specifically, it has been shown that when people are offered the option to perform one of two tasks in task switching paradigms, they typically prefer the easier task above the more difficult task [30]. As such, the offender´s preference to tell the truth rather than lie, may indicate the avoidance of cognitive effort (lying) and a preference for the easy route (telling the truth). Again, we stress that an alternative explanation is provided by the payment scheme that may have led to an avoidance of the task (lying) that was associated with an increased risk of financial loss.

### The cognitive costs of lying

When instructed to lie versus tell the truth, we found that offenders made nearly twice as many errors and were substantially slower when lying compared to when telling the truth. This indicated that the behavioral costs of lying previously observed in undergraduate and community samples ([1], [3], [29]; [31–34]), generalizes to the forensic population (see [35–36]), and suggests that lying requires more mental effort than truth telling, also in offenders. According to the Activation-Decision-Construction-Action Theory (ADCAT; [37]), the cognitive cost of lying may be related to one or more of the following cognitive components: Automatic *activation* of the truth, the *decision* to lie, the *construction* of the lie, or *acting* sincere. Given the non-interactive nature of our task, the action component is not at play. The use of instructed lying versus truth telling also excludes the decision component. This implies that the activation of the truth and/or the construction of the lie were sufficient to produce a notable behavioral cost for lying. The observed cognitive cost of lying in offenders requires qualification. First, lacking a non-offender control group, we do not know whether lying is as mentally taxing for offenders as it is for non-offenders. Second, that offenders displayed more mental effort for lying than for truth telling was observed within a very specific laboratory paradigm. We chose for the Sheffield lie task because it consistently provides maximal experimental control and leads to robust lie-truth differences. A downside of the task is its highly artificial nature, that has led to concerns with regard to whether the task still measures deception (e.g [38]). The task constraints may also have limited the offender´s ability to lie proficiently. It is worthwhile to extend the assessment of the cognitive load of lying to contexts that involve interaction with another person or situations in which the personal gains are of higher stake. Third, the greater difficulty in responding to the lie trials than to the truth trials may partly be explained by the overall higher proportion of truth trials. Recall that 1/3ed of the trials were choice trials, and that most offenders mostly chose to tell the truth. This implies that aggregating cued and choice trials, there was a higher proportion of truth trials than lie trials for most participants. A higher proportion of truth trials may facilitate truthful responding and hamper deceptive responding ([29], [33–34]). The observation in the present study that a higher lying frequency on the choice trials was related to reduced lying difficulty, supports this possibility. Alternatively, a smaller cognitive cost of lying may be associated with a greater tendency to lie. Thus, our findings should be replicated with a balanced proportion of lie and truth trials to corroborate whether indeed offenders have greater difficulty with lying than with truth telling.

### Callous-Unemotional traits and the ease of lying

The cognitive cost of lying (as assessed by error rates) appeared smaller in offenders with high callous-unemotional traits, but that correlation was carried entirely by two outliers. After excluding those with an extreme score on the YPI-CU, the correlation was no longer significant. Clearly, those two cases are highly influential. Should the outliers be excluded from the analyses? A first possibility is that the association is real, hence that the cognitive cost of lying is only reduced in those with extremely elevated callous-unemotional traits. Such an interpretation fits with a categorical view on psychopathy [39]. While our data remain inconclusive, we think that the observed correlation is most likely spurious, and that there is no real association between callous-unemotional traits and ease of lying in the present dataset. Note that the effect was apparent only in error rates and not the RTs (which typically is the more reliable and valid index of the cognitive load of deception; [3]). Moreover, despite the apparent greater ease of lying, callous-unemotional offenders still displayed a substantial cognitive cost of lying. While encouraging further research, we conclude that callous-unemotional traits, or psychopathy more generally, does not seem to present a great threat to computerized cognition-based lie detection.

### Grandiose-Manipulative traits and compulsive lying

While offenders largely preferred to tell the truth on the choice trials, there was large heterogeneity: Some offenders nearly always chose to tell the truth and some lied almost all of the time. Grandiose-manipulative traits partly explains this variability. High grandiose-manipulative offenders chose to lie 3 times as often as low grandiose-manipulative offenders. Grandiose-manipulative offenders may be compulsive liars, and lie despite probably negative consequences. Grandiose-manipulative traits were, however, not related to the cognitive costs of lying. Perhaps then, this association can be explained by poor self-judgement. Whereas they do not really have superior lying skills, grandiose-manipulative offenders seem to consider themselves as swift liars, up to the point that they engage in lying when it has probable negative consequences. While we consider the higher lying frequency on the choice trials as implying compulsive lying, we are mindful of alternative explanations. For instance, random responding (e.g., due to boredom) may alternatively explain the higher lying frequency on the choice trials. Fig 2 shows that the lie rate in the high grandiose-manipulative offenders is mostly in the 37.5%–62.5% range, which is the range expected for random responding on a binary task (lie vs tell the truth). Then again, there were no other indications (e.g., elevated error rate) of boredom in the grandiose-manipulative offenders. Still, our novel measure of compulsive lying needs further refinement to rule out alternative explanations such as random responding.

## Conclusions

Taking the limitations of our study into account, our findings generally support the cognitive view of lying. Offenders evidenced greater cognitive load for lying than for truth telling, and largely preferred to tell the truth in the face of negative consequences coupled to lying. Clinically, it is interesting that grandiose-manipulative offenders chose to lie in spite of probable negative consequences—pointing to compulsive lying. From an applied perspective, it is important to note that there was a substantial cognitive cost for lying, that did not vary with psychopathic traits. While this finding is restricted to an artificial laboratory deception paradigm and should be followed up with paradigms that involve social interaction, it does indicate that psychopathy may not be a major threat to such computerized cognition-based lie detection tests.

## Supporting Information

S1 File Psychopathy Checklist Revised (PCL-R). (DOCX)

## References

[1] Spence SA, Farrow TFD, Herford AE, Wilkinson ID, Zheng Y, Woodruff PWR. Behavioural and functional anatomical correlates of deception in humans. Neuroreport. 2001;12:2849–53. 10.1097/00001756-200109170-00019 11588589

[2] Gamer M. Detecting of deception and concealed information using neuroimaging techniques In: Verschuere B, Ben-Shakhar G, Meijer EH, editors. Memory detection: Theory and application of the concealed information test Cambridge: Cambridge University Press; 2011 p. 90–113. 10.1017/cbo9780511975196.006

[3] Verschuere B, Suchotzki K, Debey E. Detecting deception through reaction times In: Granhag PA, Vrij A, Verschuere B. Deception detection: Current challenges and new approaches. Oxford: John Wiley & Sons, Inc 2015 p. 269–291. 10.1002/9781118510001.ch12

[4] Caso L, Gnisci A, Vrij A, Mann S. Processes underlying deception: An empirical analysis of truth and lies when manipulating the stakes. J Invest Psychol Off. 2005;2:195–202. 10.1002/jip.32

[5] Vrij A, Semin GR, Bull R. Insight into behavior displayed during deception. Hum Commun Res. 1996; 22:544–562. 10.1111/j.1468-2958.1996.tb00378.x

[6] Vrij A, Fisher R, Mann S, Leal S. Detecting deception by manipulating cognitive load. Trends Cogn Sci. 2006; 10:141–142. 16516533 10.1016/j.tics.2006.02.003

[7] Vrij A, Granhag PA. Eliciting cues to deception and truth: What matters are the questions asked. J Appl Res Mem Cogn. 2012; 1:110–117. 10.1016/j.jarmac.2012.02.004

[8] Hare RD. Manual for the Hare Psychopathy Cheklist-Revised 2nd ed. Toronto: Multi-Health Systems. 1991/2003.

[9] Robbins L. Deviant children grown up. Baltimore: Williams & Wilkins 1966 10.1093/sf/45.3.464

[10] Andershed H, Kerr M, Stattin H, Levander S. Psychopathic traits in non-referred youths: Initial test of a new assessment tool In: Blaauw E, Sheridan L, editors. Psychopaths: Current international perspectives. 2002 p.131–158. The Hague: Elsevier.

[11] Hare RD, Forth AE, Hart SD. The psychopathy as prototype for pathological lying and deception In: Yuille JC, editor. Credibility Assessment. 1989 p. 25–49. Dordrecht: Kluwer Academic Publishing 10.1007/978-94-015-7856-1_2

[12] Fullam RS, Mckie S, Dolan MC. Psychopathic traits and deception: functional magnetic resonance imaging study. Br J Psychiatry. 2009; 194:229–235. 10.1192/bjp.bp.108.053199 19252152

[13] Halevy R, Shalvi S, Verschuere B. Being Honest About Dishonesty: Correlating Self-Reports and Actual Lying. Hum Commun Res. 2014; 40:54–72. 10.1111/hcre.12019

[14] Hoffman A, Diedenhofen B, Verschuere B, Musch J. A strong validation of the Crosswise Model using experimentally induced cheating behavior. Exp Psychol. 2015; 62:403–414. 10.1027/1618-3169/a000304 27120562

[15] Klaver JR, Lee Z, Spidel A, Hart SD. Psychopathy and deception detection using indirect measures. Legal Criminol Psychol. 2009; 14:171–182. 10.1348/135532508x289964

[16] Ray JV, Hall J, Rivera-Hudson N, Poythress NG, Lilienfeld SO, Morano M. The relation between self-reported psychopathic traits and distorted response styles: A meta-analytic review. Personal Disord. 2013; 4:1–14. 22452779 10.1037/a0026482

[17] Rogers R, Vitacco MJ, Jackson RL, Martin M, Collins M, Sewell KW. Faking Psychopathy? An Examination of Response Styles With Antisocial Youth. J Pers Assess. 2002; 78:31–46. 10.1207/s15327752jpa7801_03 11936210

[18] Verschuere B, Crombez G, De Clercq A, Koster EHW. Psychopathic traits and autonomic responding to concealed information in a prison sample. Psychophysiology. 2005; 42:239–245. 10.1111/j.1469-8986.2005.00279.x 15787861

[19] Verschuere B, Crombez G, De Clercq A, Koster E. Antisociality, underarousal, and the validity of the Concealed Information polygraph Test. Biol Psychol. 2007; 74:309–318. 10.1016/j.biopsycho.2006.08.002 17030398

[20] Spidel A, Hervé H, Greaves C, Yuille JC. ‘Wasn’t me!’ A field study of the relationship between deceptive motivations and psychopathic traits in young offenders. Legal Criminol Psychol. 2011; 16:193–384.

[21] Hall J, Benning S. The "successful" psychopath: Adaptive and subclinical manifestations of psychopathy in the general population In: Patrick CJ, editor. Handbook of psychopathy. New York: Guilford Press 2006 p. 459–478.

[22] Lilienfeld SO, Watts AL, Smith SF. Successful Psychopathy A Scientific Status Report. Curr Dir Psychol Sci. 2015; 24:298–303. 10.1177/0963721415580297

[23] Conradi HJ, Boertien SD, Cavus A, Verschuere B. Examining psychopathy from an attachment perspective: The role of fear of rejection and abandonment. J Forens Psychiatry Psychol. 2015 10.1080/14789949.2015.1077264

[24] Hillege S, Das J, de Ruiter C. The Youth Psychopathic traits Inventory: Psychometric properties and its relation to substance use and interpersonal style in a Dutch sample of non-referred adolescents. J Adolesc. 2010; 33:83–91. 10.1016/j.adolescence.2009.05.006 19559475

[25] Uzieblo K, Verschuere B, Van den Bussche E, Crombez G. The Validity of Psychopathy Personality Inventory—Revised in a Community Sample. Assessment. 2010; 17:334–346. 10.1177/1073191109356544 20040727

[26] Inquisit 4.00 [Computer software]; 2013. Seattle, WA: Millisecond Software.

[27] Edens JF, Marcus DK, Lilienfeld SO, Poythress NG Jr. Psychopathic, not psychopath: taxometric evidence for the dimensional structure of psychopathy. J Abnorm Psychol. 2006; 115:131 16492104 10.1037/0021-843X.115.1.131

[28] Greenwald AG, Nosek BA, Banaji MR. Understanding and using the implicit association test: I. An improved scoring algorithm. J Pers Soc Psychol. 2003; 85:197–216. 12916565 10.1037/0022-3514.85.2.197

[29] Verschuere B, Spruyt A, Meijer EH, Otgaar H. The ease of lying. Conscious Cogn. 2011; 20:908–911. 10.1016/j.concog.2010.10.023 21093302

[30] Arrington CM, Logan GD. The cost of a voluntary task switch. Psychol Sci. 2004; 15:610–615. 10.1111/j.0956-7976.2004.00728.x 15327632

[31] Debey E, Verschuere B, Crombez G. Lying and executive control: An experimental investigation using ego depletion and goal neglect. Acta Psychol. 2012; 140: 133–141. 10.1016/j.actpsy.2012.03.004 22627157

[32] Spence SA, Kaylor-Hughes CJ. Looking for truth and finding lies: The prospects for a nascent neuroimaging of deception. Neurocase. 2008; 14:68–81. 10.1080/13554790801992776 18569733

[33] Van Bockstaele B, Verschuere B, Moens T, Suchotzki K, Debey E, Spruyt A. Learning to lie: Effects of practice on the cognitive cost of lying. Front Psychol. 2012; 3:526 10.3389/fpsyg.2012.00526 23226137 PMC3510470

[34] Van Bockstaele B, Wilhelm C, Meijer E, Debey E, Verschuere B. When deception becomes easy: The effects of task switching and goal neglect on the truth proportion effect. Front Psychol. 2015; 6:1666 10.3389/fpsyg.2015.01666 26579047 PMC4630537

[35] Jiang W, Liu H, Liao J, Ma X, Rong P, Tang Y, et al A functional MRI study of deception among offenders with antisocial personality disorders. Neuroscience. 2013; 244:90–98. 10.1016/j.neuroscience.2013.03.055 23578713

[36] Kaylor-Hughes CJ, Lankappa ST, Fung R, Hope-Urwin AE, Wilkinson ID, Spence SA. The functional anatomical distinction between truth telling and deception is preserved among people with schizophrenia. Crim Behav Ment Health. 2011; 21:8–20. 10.1002/cbm.785 20661881

[37] Walczyk JJ, Harris LL, Duck TK, Mulay D. A social-cognitive framework for understanding serious lies: Activation—decision—construction—action theory. New Ideas Psychol. 2014; 34:22–36. 10.1016/j.newideapsych.2014.03.001

[38] Sip KE, Roepstorff A, McGregor W, Frith CD. Detecting deception: the scope and limits. Trends Cogn Sci. 2007; 12:48–53. 10.1016/j.tics.2007.11.008 18178516

[39] Brazil IA. Considering new insights into antisociality and psychopathy. Lancet Psychiatry. 2015; 2:115–116. 10.1016/s2215-0366(14)00125-4 26359737

