# Supplementary material for: Psychopathic Traits and Their Relationship with the Cognitive Costs and Compulsive Nature of Lying in Offenders
Source: PLoS One. 2016 Jul 8;11(7):e0158595. doi: 10.1371/journal.pone.0158595 (PMC4938600; doi:10.1371/journal.pone.0158595)
Supplement: S1 File — (DOCX) [file pone.0158595.s001.docx]

**Psychopathy Checklist Revised (PCL-R)**

PCL-R scores were available for 49 patients. Unfortunately, we lacked PCL-item scores, preventing to calculate PCL-R factor scores for the 3-/4-factor solution and to calculate the internal consistency of the PCL-R facet/factor scores. Also, no interrater reliability index could be calculated. For sake of completion we report here upon the association between the PCL-R and the lying indices.

**Method**

The Psychopathy Checklist-Revised (PCL-R; Hare, 2003; Dutch translation: Vertommen, Verheul, de Ruiler, & Hildebrand, 2002) consists of 20-items characteristic of psychopathy that are scored on their presence (0 = absent; 1 = sometimes/perhaps; 2 = present) based upon a structured clinical interview and file review. PCL-R scores can vary from 0 to 40. The PCL-R is an obligatory part of risk assessment in TBS patients residing in Dutch forensic psychiatric hospitals. Previous studies have shown high inter-rater reliability and validity for the Dutch version of the PCL-R (see e.g., Zwets et al., in press). In the present study we used the most recent PCL-R score available, which was typically based upon a consensus between two experienced forensic psychologists, trained in the background and use of the PCL-R through a 3-day PCL-R workshop held in the Netherlands. From the patient´s file, we could retrieve the PCL-R total score as well as the PCL-R Factor scores of the original 2-factor solution (Harpur, Hakstian, & Hare, 1988): PCL-R Factor1 encompassing affective-interpersonal items (e.g., lack of empathy, glibness/superficial charm), and PCL-R Factor2 encompassing behavioral-lifestyle items (e.g., poor behavioral control, juvenile delinquency). While this 2-factor structure has been thoroughly investigated and validated, the PCL-R factor structure remains debated with some authors proposing a 3-factor solution (Cooke & Michie, 2001) and others proposing a 4-factor solution (Hare, 2003).

**Descriptive statistics**

*M*_PCL-R Total score_ = 23.90; *SD* = 7.15; range: 7-37. PCL-R scores were unrelated to age, *r*_PCL-R, age_= .00, *p* = .99; and IQ *r*_PCL-R, IQ_= -.17, *p* = .28.

**Associations with lying indices**

There was no significant relation between the PCL-R and the cognitive cost of lying nor compulsive lying, see Table 2 below.

Table 2. Correlation matrix displaying the associations between the PCL-R and the cognitive costs of lying (in RTs and errors), and lying frequency on the choice trials. This is Table 2 legend.

|  | P C L - R | | D e c e p t i o n I n d i c e s | | |
| --- | --- | --- | --- | --- | --- |
|  | PCL-R  F2 | PCL-R  TOT | RT_LIE_-RT_TRUTH_ | Errors_LIE_-Errors_TRUTH_ | Choice to lie |
| PCL-R-F1 | .30* | .77** | .12 | .03 | .02 |
| PCL-R-F2 | - | .80*** | .18 | .19 | -.09 |
| PCL-R-TOT |  | - | .16 | .14 | .00 |

Note: Significant effects with *p*< .05 are designated as (*), effects with *p* < .01 are designated as (**), and effects with *p* < .001 are designated as (***). No correction for multiple testing was applied, and effect sizes are more important than significance levels (*r*´s can be labelled as small, moderate, and large effects, from .1, .3, and .5 onwards, respectively)

To illustrate, even the 13 offenders with a PCL-R score of 30 or higher (Hare, 2003) displayed a significant and large cognitive cost for lying compared to truth telling in both errors, *t*(12) = 3.83, *p* < .01, *d_within_* = 1.06 (95% CI: -0.16 – 2.28), and RTs, *t*(12) = 3.69, *p* < .01, *d_within_* = 1.03 (95% CI: 0.67 – 1.39).
